# The Lipid Interactome: an interactive and open access platform for exploring cellular lipid–protein interactions

**DOI:** 10.1093/bioinformatics/btaf651

**Published:** 2026-02-18

**Authors:** Gaelen Guzman, André Nadler, Frank Stein, Jeremy M Baskin, Carsten Schultz, Fikadu G Tafesse

**Affiliations:** Department of Molecular Immunology & Immunology, Oregon Health & Science University, Portland, OR 97239, United States; Max Plank Institute of Molecular Cell Biology and Genetics, 01307 Dresden, Germany; Proteomics Core Facility, European Molecular Biology Laboratory, 69117, Heidelberg, Germany; Department of Chemistry and Chemical Biology and Weill Institute for Cell and Molecular Biology, Cornell University, Ithaca, NY 14853-1301, United States; Department of Chemical Physiology & Biochemistry, Oregon Health & Science University, Portland, OR 92739, United States; Department of Molecular Immunology & Immunology, Oregon Health & Science University, Portland, OR 97239, United States; Department of Chemical Physiology & Biochemistry, Oregon Health & Science University, Portland, OR 92739, United States

## Abstract

**Summary:**

Lipid–protein interactions play essential roles in cellular signaling and membrane dynamics, yet their systematic characterization has long been hindered by the inherent biochemical properties of lipids. Recent advances in functionalized lipid probes—equipped with photoactivatable crosslinkers, affinity handles, and photocleavable protecting groups—have enabled proteomics-based identification of lipid interacting proteins with unprecedented specificity and resolution. Despite the growing number of published lipid interactomes, there remains no centralized effort to harmonize, compare, or integrate these datasets. The Lipid Interactome addresses this gap by providing a structured, interactive web portal that adheres to FAIR data principles—ensuring that lipid interactome studies are Findable, Accessible, Interoperable, and Reusable. Through standardized data formatting, interactive visualizations, and direct cross-study comparisons, this resource enables researchers to systematically explore the protein-binding partners of diverse bioactive lipids. By consolidating and curating lipid interactome proteomics data from multiple studies, the Lipid Interactome database serves as a critical tool for deciphering the biological functions of lipids in cellularsystems.

**Availability and implementation:**

This site can be viewed at LipidInteractome.org. All data are available for download. No user information is collected or necessary for data navigation, interaction, or download.

## 1 Introduction

Historically, lipids have been considered merely the passive structural components of cellular membranes. However, recent decades have revealed that this diverse category of biomolecule plays essential and active roles in the cell, ranging from modulating membrane dynamics, determining organelle identity, serving as catabolites for energy storage and metabolism, and bioactive signaling mediators for organellar and intracellular signaling (Chernomordik and Kozlov 2003, van Meer *et al.* 2008, Holthuis and Menon 2014, Niphakis *et al.* 2015, Barisch *et al.* 2023, Simons and Toomre 2000). Interactions with proteins are essential to many of these crucial lipid functions—and yet, lipid–protein interactions are extremely challenging to study: The small size and biophysical properties of lipids make conventional proteomics approaches unsuitable for mapping lipid interactors (Schultz 2023). Unlike proteins, which can be readily tagged or immobilized for affinity-based interaction studies, lipids require specialized chemical modifications to enable selective and controlled interaction profiling (Schultz *et al.* 2022).

Recent advances in synthetic, multifunctionalized lipid analogs have revolutionized the field, enabling the systematic interrogation of lipid interactomes (Schultz *et al.* 2022). These probes incorporate strategically designed functional groups that enable covalent crosslinking, affinity enrichment, and controlled activation. Most commonly, these include: (i) a diazirine group for photoactivatable crosslinking upon exposure to 355 nm ultraviolet (UV) light, stabilizing non-covalent and often modest-affinity lipid–protein interactions; (ii) a terminal alkyne, permitting click chemistry-based enrichment; and, in some cases, (iii) a coumarin photocage, which shields the lipid probe from premature enzymatic modification until it is selectively cleaved by 405 nm light (a wavelength that does not induce diazirine photolysis) (Haberkant and Holthuis 2014, Höglinger *et al.* 2017, Schultz *et al.* 2022). Together, these modifications enable direct proteomic identification of lipid interactors, distinguishing true binding partners from transient or non-specific associations (Schultz 2023, Farley *et al.* 2024, Haberkant and Holthuis 2014).

Numerous studies have already used these lipid probes to elucidate the interactomes of key bioactive lipids, including diacylglycerol, sphingosine, sphinganine, phosphatidic acid, and phosphatidylethanolamine, N-acylphosphatidylethanolamine, and phosphatidyl alcohol (Höglinger *et al.* 2017, Müller *et al.* 2021, Yu *et al.* 2022, Farley *et al.* 2024a,b, Chiu *et al.* 2025, Thomas *et al.* 2025). These initial studies have followed a comparative proteomics workflow, wherein crosslinked (“+UV”) and non-crosslinked (“−UV”) samples are analyzed side by side to minimize artifacts and confirm specificity (Haberkant and Holthuis 2014, Schultz 2023). However, despite the growing number of lipid interactome datasets, no centralized resource has existed to compile, compare, and standardize these findings.

To address this gap, we present the Lipid Interactome—a curated repository designed to facilitate the comparative analysis of lipid–protein interactions from multiple studies. This resource consolidates proteomics data from multifunctional lipid probes, enabling researchers to efficiently explore and contextualize lipid-binding proteins across different lipid species and cellular models. Currently, the repository hosts interactomes derived from trifunctionalized phosphatidic acid, phosphatidylethanolamine, phosphatidylinositol *tris*phosphate, *N*-acylphosphatidylethanolamine, phosphatidyl alcohol, sphingosine, and sphinganine in HeLa and Huh7 cells (Müller *et al.* 2021, Yu *et al.* 2022, Farley *et al.* 2024a, Chiu *et al.* 2025, Thomas *et al.* 2025). Additionally, it includes control datasets from two fatty acid probes to distinguish specific from non-specific lipid-binding proteins (Farley *et al.* 2024b). We additionally intend to expand this resource to include more complex experimental designs as they are published (e.g. Disease versus Healthy Control or Infection versus Mock).

By providing a centralized platform for lipid interactome data, the Lipid Interactome significantly lowers the barriers to entry for researchers investigating lipid signaling pathways, enabling hypothesis generation through comparative analysis of multiple studies. Overall, this repository represents an important step toward systematizing lipid–protein interaction studies, ensuring that bioactive lipids are afforded the same level of proteomic scrutiny as their protein counterparts.

## 2 Description

### 2.1 Overview

The Lipid Interactome is an interactive discovery platform built using Quarto, designed to centralize and enhance the study of lipid–protein interactions through curated datasets and dynamic visualization tools. Each page integrates R-generated figures, embedding scripts directly into the source material to ensure reproducibility and transparency. The repository currently hosts data from seven published studies, with an expandable framework that supports the incorporation of future datasets as the field progresses.

At its core, the Lipid Interactome enables the direct, qualitative comparison of lipid interactomes between studies, providing researchers with invaluable insights into the diverse roles that bioactive lipids play in cellular processes. By leveraging standardized data formatting and interactive visualization—including study-specific analysis pages, lipid probe aggregation, and cross-study comparisons via a Shiny application—the platform facilitates intuitive exploration of protein–lipid interactions. As a living repository, we actively encourage researchers to contribute their findings through our Data Submission page, ensuring that this resource continues to evolve alongside advancements in lipidomics research.

### 2.2 FAIR data principles

The Lipid Interactome database was developed with the FAIR (Findable, Accessible, Interoperable, and Reusable) data principles in mind, ensuring that lipid–protein interaction data can be systematically explored, compared, and reused by the research community. Given the historical inconsistency in proteomics data curation, we have taken deliberate steps to standardize, annotate, and document our datasets for maximum utility.

#### 2.2.1 Findable

To address the fragmented nature of lipid interactome studies, we have established a centralized platform where researchers can interact with the results of individual studies or explore aggregated findings across multiple studies on a given lipid. The repository is structured to facilitate intuitive navigation between experimental datasets, offering clear entry points for study-specific and lipid-specific analyses. Because this resource is intended as a living repository, we actively invite researchers to contribute their findings following the submission guidelines detailed on our Data Submission page.

#### 2.2.2 Accessible

All datasets within the Lipid Interactome are available for download in standardized .csv format, ensuring broad accessibility across different computational environments. To further enhance usability, we provide detailed overviews of data generation and analysis, including explicit descriptions of dataset structure, column definitions, and the inherent limitations of multifunctional lipid probes. Only peer-reviewed proteomics datasets are included in this repository to maintain high data quality and reliability.

#### 2.2.3 Interoperable

Given the diversity of methodologies used across lipid interactome studies, we have taken care to harmonize data formats and metadata annotations. While many studies share common analytical pipelines, others use distinct mass spectrometry workflows. To ensure consistency, we have structured the data to allow parallel comparisons and provided documentation on methodological differences. This standardization effort facilitates direct cross-study comparisons without requiring extensive reformatting by the end user. Each dataset is accompanied by metadata which describe the sample handling and data analysis techniques—with clarifying text describing how “significance” is determined for each analysis.

However, we emphasize that the datasets presented on this site have not been fully harmonized at present: differences in mass spectrometer instrumentation, sample handling, and statistical testing used by each publication are irreconcilably distinct—thus, fold-change and *P* values between studies are not compatible with quantitative comparisons. For this reason, we do not report fold-change enrichment between individual lipids reported on this site and produce only visualizations of the comparisons presented in the Probe versus Probe Comparisons Shiny app.

#### 2.2.4 Reusable

To maximize the utility of these datasets, we provide extensive documentation detailing data acquisition, analysis workflows, and potential limitations. Wherever possible, technical jargon has been minimized to ensure accessibility to a broad audience, from lipid biologists to computational scientists. Additionally, we reference key publications that delve deeper into the synthesis of lipid probes and the specifics of each proteomics workflow, allowing researchers to further contextualize and validate their findings.

By adhering to these FAIR principles, the Lipid Interactome database aims to serve as a robust and enduring resource for the lipid biology community, reducing redundancy, improving data transparency, and accelerating the study of lipid-mediated signaling.

### 2.3 Data visualization and download modules

To facilitate efficient exploration and analysis of lipid–protein interaction data, the Lipid Interactome repository incorporates a suite of interactive visualization and data access tools. These modules are designed to enhance user engagement, provide clear interpretability of experimental findings, and streamline data retrieval for further computational analysis—overall, the site is designed to ensure that the Lipid Interactome is not merely a static repository, but rather a dynamic and evolving resource for lipid biologists and bioinformaticians alike.

#### 2.3.1 Study-specific data exploration

Each publication contributing to the repository is provided with a dedicated results page, allowing users to examine study findings in detail. These pages include interactive Volcano, Ranked-Order, and MA plots, which enable visualization of differential protein enrichment across experimental conditions. The plots are rendered using the Plotly and htmlwidgetsR packages, ensuring a responsive, client-side experience that supports zooming, hovering, image export, and data filtering (Sievert *et al.* 2015, Ramnath Vaidyanathan *et al.* 2015). For transparency and reproducibility, all datasets associated with a given study are available for direct download.

#### 2.3.2 Lipid probe-specific pages

In addition to the pages for each independent publication, datasets are further organized by lipid probe, providing a consolidated view of protein interactors detected across multiple experiments and independent studies. Each lipid-specific page includes study metadata and cell type—facilitating cross-study comparisons. Future iterations of the repository will extend this functionality by enabling direct comparisons of interactomes obtained from different cell lines treated with the same lipid probe, further refining the contextual understanding of lipid–protein interactions.

#### 2.3.3 Comparative analysis via Shiny application

Where study design permits, data has been integrated into an interactive Shiny application, allowing users to perform direct (though qualitative) probe-to-probe comparisons (Probe versus Probe Comparisons). This tool enables researchers to explore the similarity of lipid interactomes across experimental conditions via linear regression analysis, highlighting proteins that are significantly enriched in one dataset, both datasets, or neither. By providing an intuitive framework for interactome comparison, this feature aids in the identification of promising targets for further investigation.

Because each publication utilizes distinct sample handling and data analysis techniques, we emphasize that these comparisons are qualitative—we hope to discourage quantitative statements regarding relative enrichment between lipid probes (e.g. “Protein X is enriched 3x more to Lipid A than to Lipid B”). However, great utility lies in assessing co-enrichment (or orthogonal enrichment) of proteins to disparate lipid probes without quantitative comparison.

### 2.4 Data submission format

While there are numerous ways to quantify the mass spectrometry (MS) data, the majority of studies included in our repository have utilized the same (or highly similar) quantitative MS pipeline using data-dependent acquisition techniques, such as Tandem Mass Tagging (Thompson *et al.* 2003). These datasets include those reported by (Farley *et al.* 2024a,b, Thomas *et al.* 2025). Some studies have also featured quantitative MS analysis using stable isotope labeling of amino acids in cell culture (SILAC), which also enables extraction of key data described below (Yu *et al.* 2022, Chiu *et al.* 2025). Correspondingly, the ideal data structure for incorporation into our repository will contain the information shown in Table 1, and includes isoform-specific protein identifications, enrichment values against a control condition, and statistical outputs such as *P* value and false-discovery rate.

**Table 1.** Example data table for smooth incorporation into Lipid Interactome.^a^

Gene name	Uniprot accession	logFC	*P* value	AveExpr	FDR
GeneA	P80723	1.2	0.001	1.0×10^18^	0.0001
GeneB	P32004	0.1	0.6	1.0×10^5^	0.005
…	…	…	…	…	…
GeneZ	Q12772	0.5	0.2	1.0x10^8^	0.001

a Column descriptions: Gene name: the identifier for genes identified in the dataset (or Accession Number). Uniprot accession: stable identifiers of UniProtKB entries; integrates species and isoform data on the identified protein. Enrichment value: fold change of Experimental versus Control fold-change, typically log2-transformed—ideally the product of TMT or other quantitative mass spectrometry methods. *P* value: the results of an ANOVA or LIMMA analysis, corrected for multiple hypothesis testing. AveExpr: an averaged value of the *m*/*z* ion intensity of the Experimental condition and Control condition used for the enrichment value (used to prepare MA plots for quality control). FDR: false-discovery rate of protein identification. (Ellipsis is intended to imply additional proteins in this example dataset).

We wish to emphasize that we will collaborate with data providers to prepare visualizations which best suit their datasets—the example dataset above merely depicts an ideal case which allows for easy incorporation and cross-study comparison. As proteomics techniques have improved rapidly in recent years, we have done our best to incorporate many data structures into this repository.

Additionally, we are working on providing visualization functionality for studies which utilized Peptide Spectral Matching (PSM) to report enrichment toward lipid probes. Similarly, data-independent acquisition (DIA) is a quickly emerging alternative to data-dependent acquisition (DDA, e.g. TMT) (Birhanu 2023, Boys *et al.* 2023); as with PSM datasets, we are working to incorporate DIA data formats.

At present, we are accepting datasets as analyzed by the original researchers, including protein identifications, enrichment quantification, and the results of statistical testing. Summaries of both source and data wrangling can be found in the *Study Information* dropdown section of each data page. Further, the raw data (as provided directly by authors or pulled from publicly available datasets) and data wrangling scripts are available for download in the same dropdown tabs. For transparency, we have included download links to the original data and R scripts used for data wrangling.

### 2.5 Discussion

The advent of functionalized lipid probes has fundamentally transformed lipid biology by enabling the proteomic identification of lipid-binding proteins with unprecedented specificity. However, as the number of published lipid interactome studies grows, the lack of a centralized framework for comparing and integrating these datasets presents a significant challenge for deciphering biological functions of individual lipids and lipid-binding proteins holistically and identifying trends across datasets. The Lipid Interactome was developed to address this need by providing researchers with a structured platform to explore, cross-reference, and build upon previous findings. By aggregating and standardizing lipid–protein interaction data, this resource facilitates the rapid identification of candidate lipid-binding proteins and supports orthogonal validation efforts.

Designed as a continuously expanding repository, the Lipid Interactome will evolve alongside advances in lipid probe technologies and interactomics. Ongoing efforts seek to incorporate enhanced scalability of this site, more robust data storage methods, and automated data submission. These functionalities will improve the utility of this repository, enabling applications such as machine learning for pattern recognition as the site grows. Future iterations will incorporate validation studies for each lipid probe, offering researchers a dedicated space to compare findings across experimental contexts. This functionality will not only reduce redundancy in experimental design but also strengthen the reproducibility and biological interpretation of lipid interactome research.

To support this vision, we invite researchers to contribute their datasets via our Data Submission page. Using the FAIR data principles, we hope to foster a collaborative and transparent approach to lipid biology.

## Data Availability

All Lipid Interactome datasets are freely available for download on each data page, and source material for the site can be found at our GitHub repository (https://github.com/Tafesse-Lab/LipidInteractomics_Website.git).
